# Supplementary material for: The Rho-GEF PIX-1 directs assembly or stability of lateral attachment structures between muscle cells
Source: Nat Commun. 2020 Oct 6;11:5010. doi: 10.1038/s41467-020-18852-4 (PMC7538588; doi:10.1038/s41467-020-18852-4)
Supplement: Supplementary file 1 — Supplementary Information [file 41467_2020_18852_MOESM1_ESM.pdf]

Supplementary Materials for:

The Rho-GEF PIX-1 Directs Assembly or Stability of Lateral Attachment Structures Between Muscle Cells

Jasmine C. Moody, Hiroshi Qadota, April R. Reedy, C. Denise Okafor, Niveda Shanmugan, Yohei Matsunaga, Courtney J. Christian, Eric A. Ortlund, and Guy M. Benian

Supplementary Figure 1

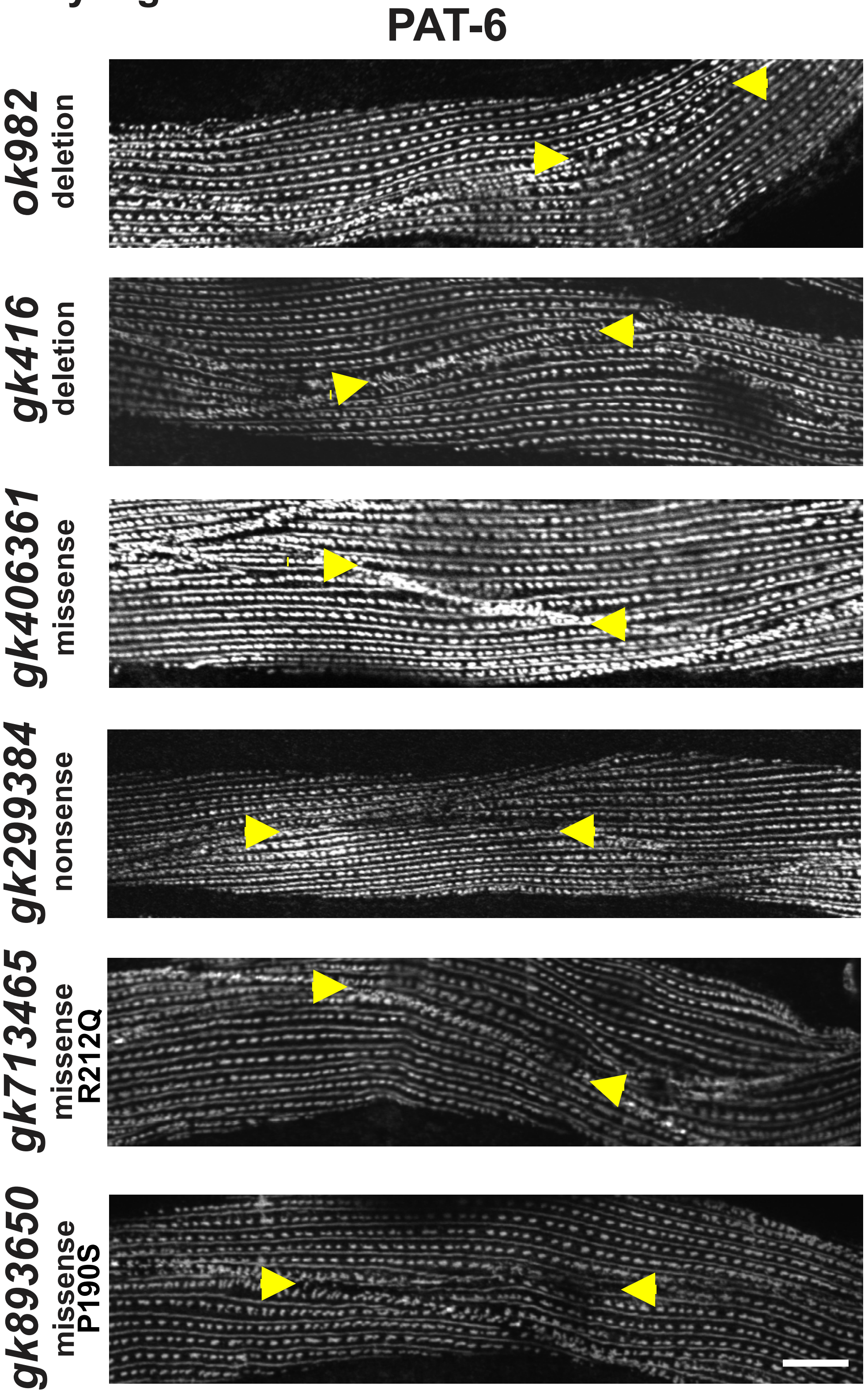

Supplementary Figure 1. Immunostaining of 6 *pix-1* mutant alleles using antibodies to PAT-6. The results are summarized in Figure 1b. Each image is a representative image obtained from at least two fixation and immunostaining experiments and imaging at least three different animals. Scale bar, 10 μm.

Supplementary Figure 2

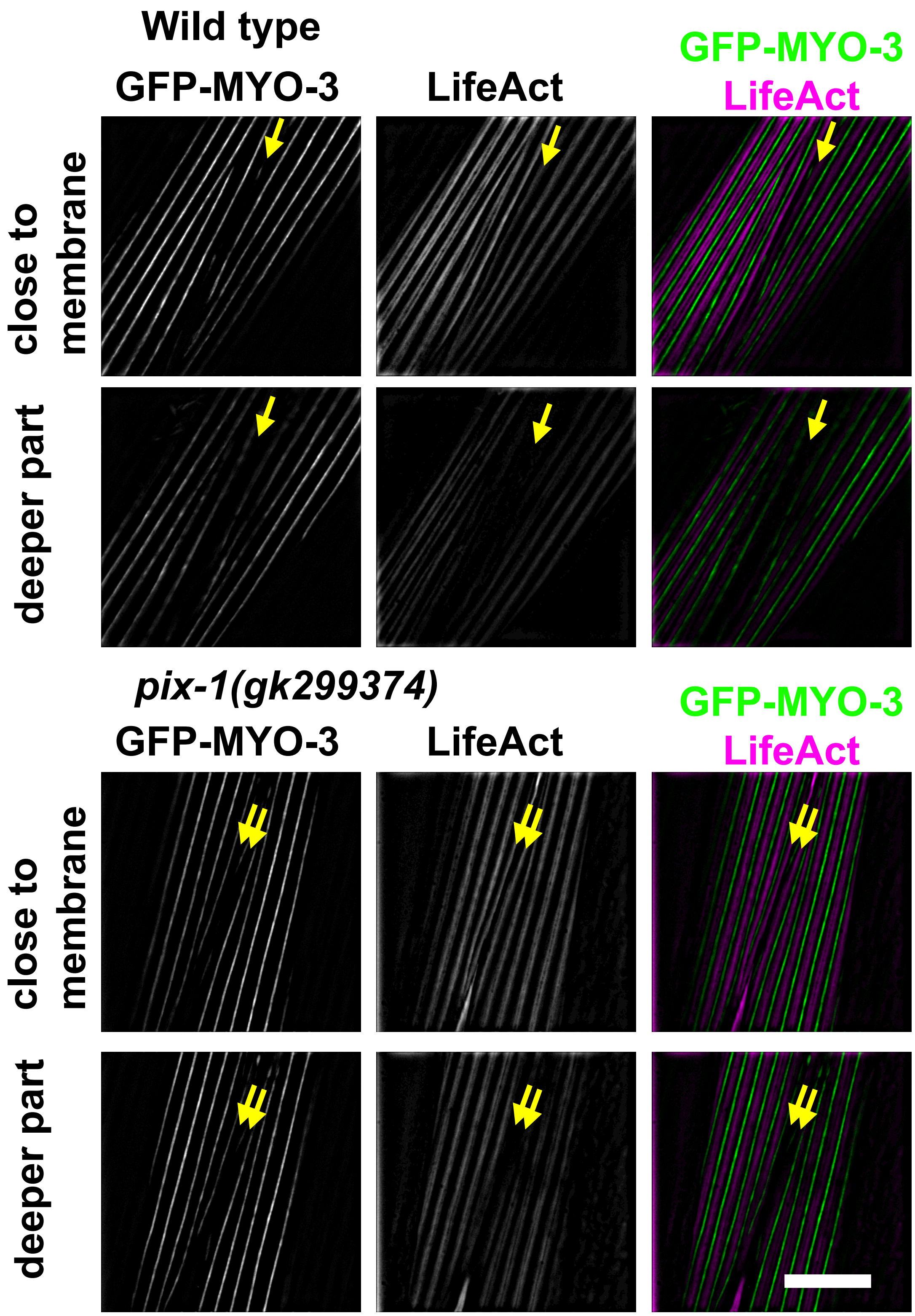

Supplementary Figure 2. Live imaging of cortical F-actin at muscle cell boundaries. SIM images of portions of two adjacent body wall muscle cells from a nematode strain in which a muscle myosin was tagged by GFP by CRISPR and LifeAct-mCherry was expressed in muscle cells from a transgene. GFP-MYO-3 labels the middle of sarcomeric A-bands, and LifeAct-mCherry labels I-bands, except for F-actin at the boundary between two adjacent muscle cells (indicated by yellow arrows). The signal from the F-actin at the boundary diminishes as the focal plane changes from close to the outer muscle cell membrane to deeper into the myofilament lattice whereas the F-actin signals from I-bands does not change. In the *pix-1* nonsense mutant, *gk299374*, there are two bands of cortical F-actin at the boundary. Each image is a representative image obtained from at least two fixation and immunostaining experiments and imaging at least three different animals.Scale bar, 10 μm. These images are the same as shown in Figure 2, except that they are shown here at lower magnification. This broader perspective allows better observation of the spindle shape of the body wall muscle cells and thus the location of the boundary between these cells.

Supplementary Figure 3

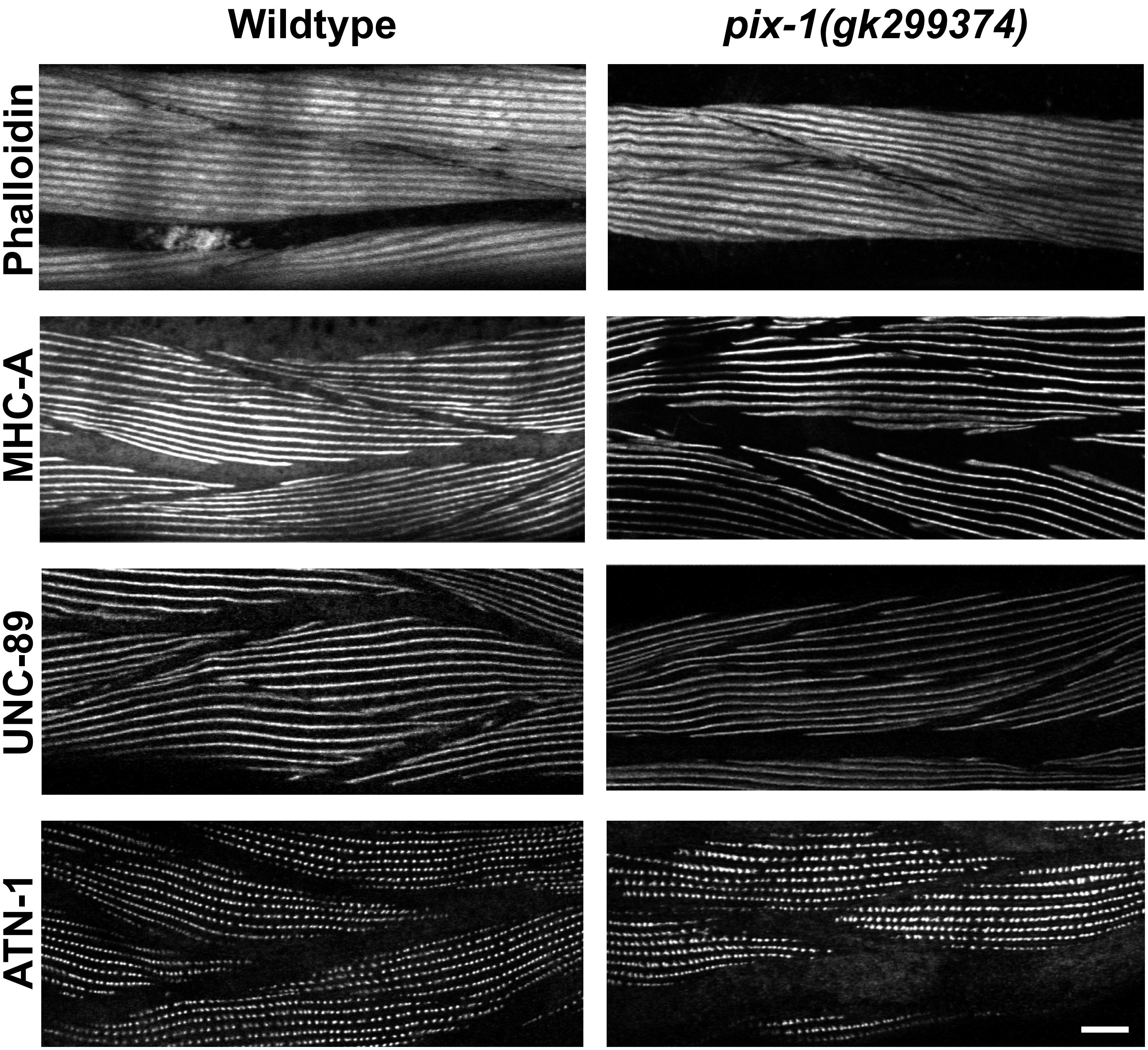

Supplementary Figure 3. A pix-1 mutant has normally-organized sarcomeres. Confocal microscopy of wild type and *pix-1(gk299374)* reacted with rhodamine-phalloidin (thin filaments), and antibodies to sarcomere proteins MHC-A (thick filaments), UNC-89 (M-lines), and ATN-1(dense bodies). Note that with each of these markers, *pix-1(gk299374)* appears the same as wild type. Each image is a representative image obtained from at least two fixation and immunostaining experiments and imaging at least three different animals. Scale bar, 10 μm.

Supplementary Figure 4

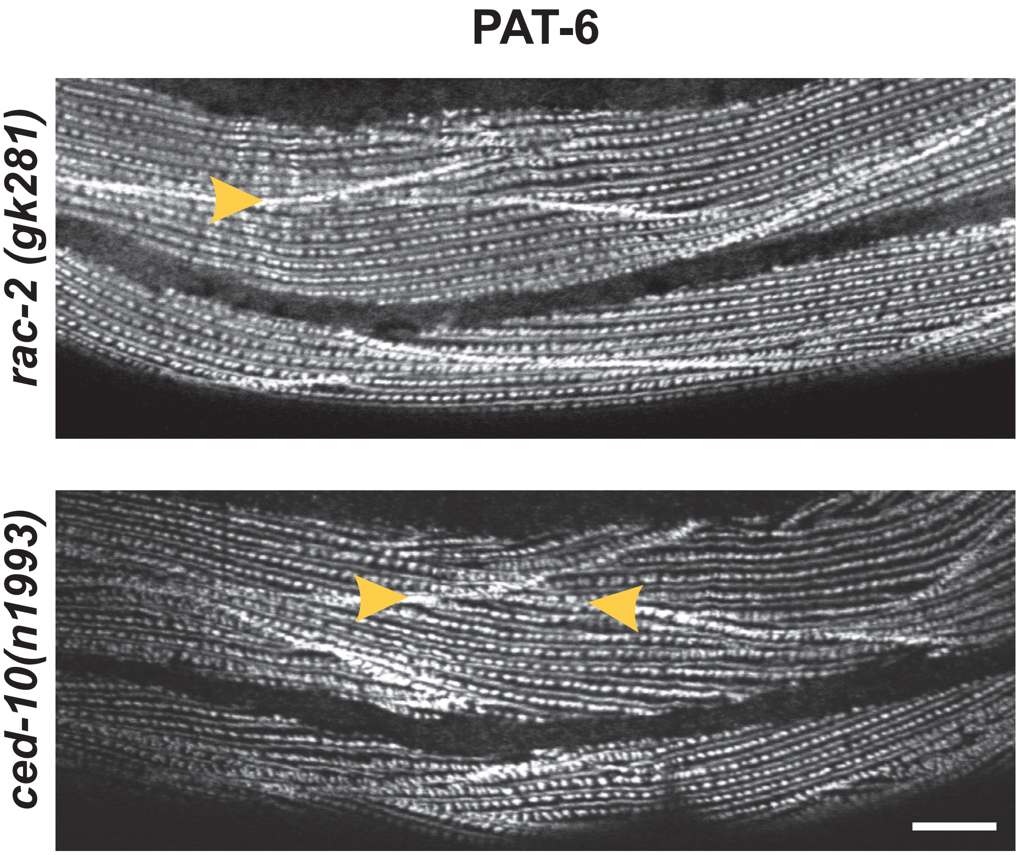

Supplementary Figure 4. PAT-6 immunostaining of two additional alleles of rac-2 and ced-10. Note that the muscle cell boundaries appear normal in *rac-2(gk281)*, in agreement with the result presented in Figure 5b on *rac-2(ok326)*. The disruption of PAT-6 organization at muscle cell boundaries is less severe for *ced-10(n1993)* shown here in comparison to *ced-10(n3246)* (shown in Figure 5b). Each image is a representative image obtained from at least two fixation and immunostaining experiments and imaging at least three different animals. Scale bar, 10 μm.

Supplementary Figure 5

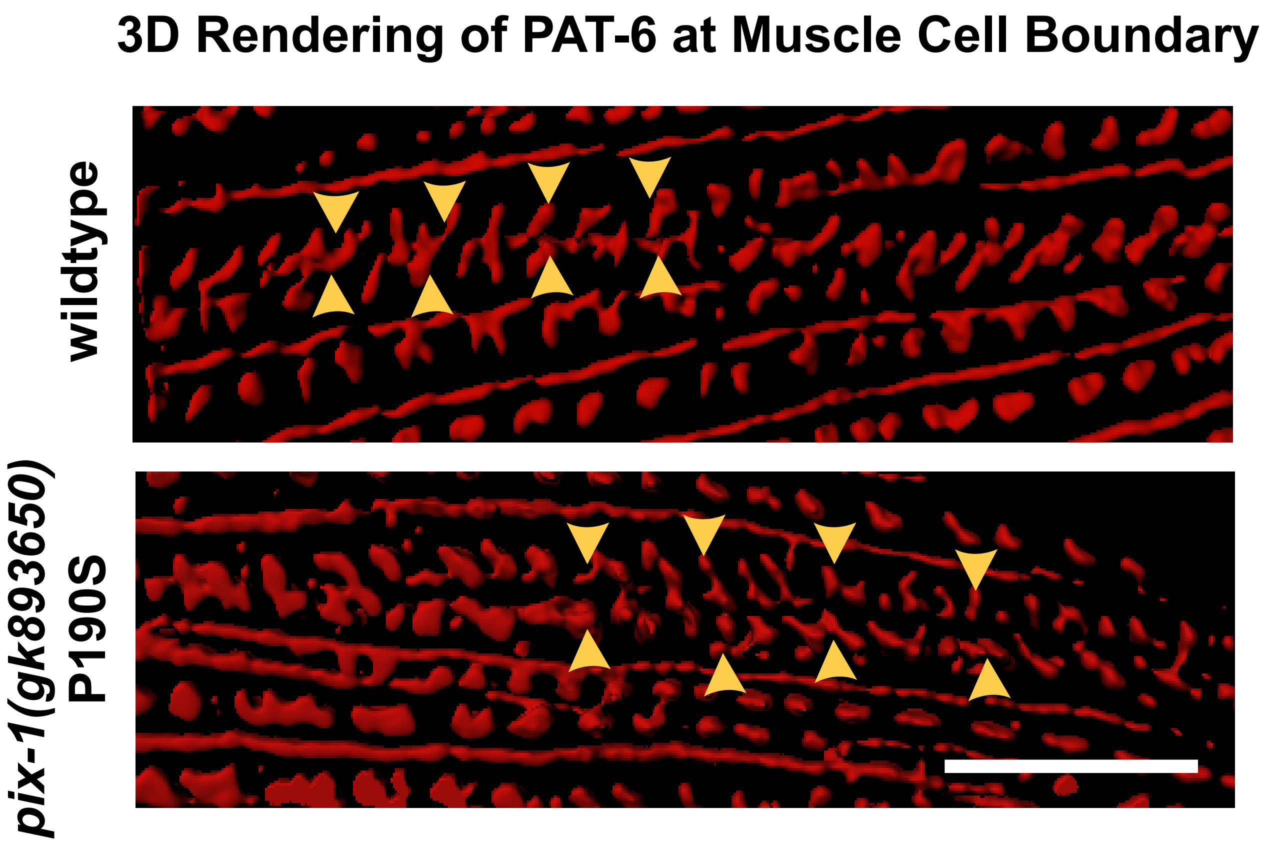

Supplementary Figure 5. 3D rendering of SIM images of PAT-6 muscle boundary localization of wild type compared with *pix-1(gk893650)*. The higher resolution of SIM compared to confocal allows clear delineation of the two sides of the “zipper-like” structure at the muscle cell boundary (arrowheads pointing down and up). In wild type the two sides of the zipper appear close together, whereas in *pix-1(gk893650)* P190S, although both sides of the zipper are present, they are more separated. Each image is a representative image obtained from at least two fixation and immunostaining experiments and imaging at least three different animals. Scale bar, 5 μm.

Supplementary Figure 6

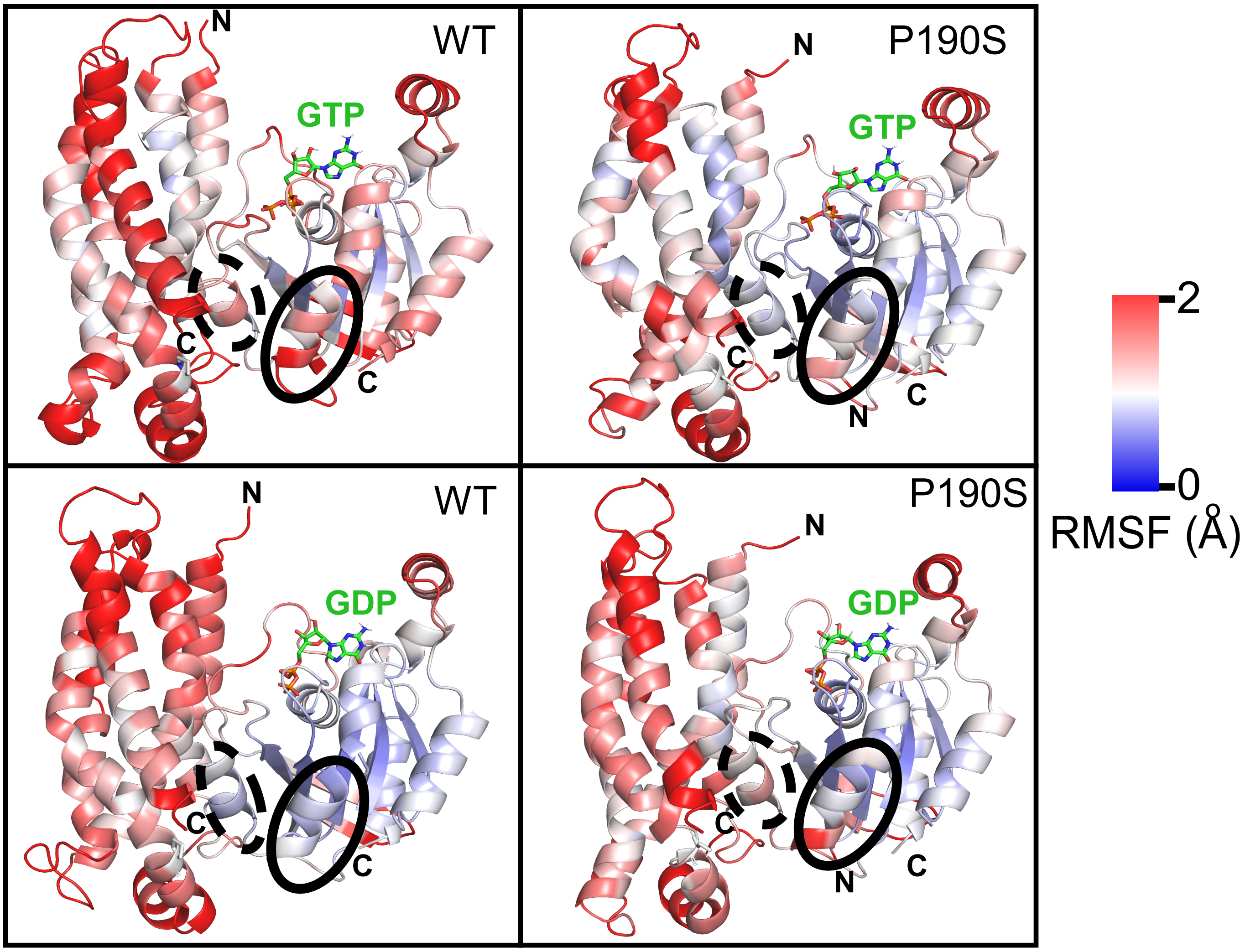

Supplementary Figure 6. Root mean square fluctuation analysis (RMSF) of PIX-1-Rac complexes indicate that the P190S mutation alters stability at the interface. Proteins are colored by RMSF as indicated. All regions colored red indicate RMSF > 2 angstroms over the simulation. To describe fluctuations on the interface, we focused on the indicated helical segments (dashed line = PIX-1, solid line = Rac) that are predicted to be in contact. In GTP-Rac complexes, the P190S mutation stabilizes the interaction between the two helices, as RMSF values on the two helices decrease. In GDP-Rac complexes, RMSF values show a net increase, indicating that the P190S mutation increases fluctuations compared to WT.

Supplementary Figure 7

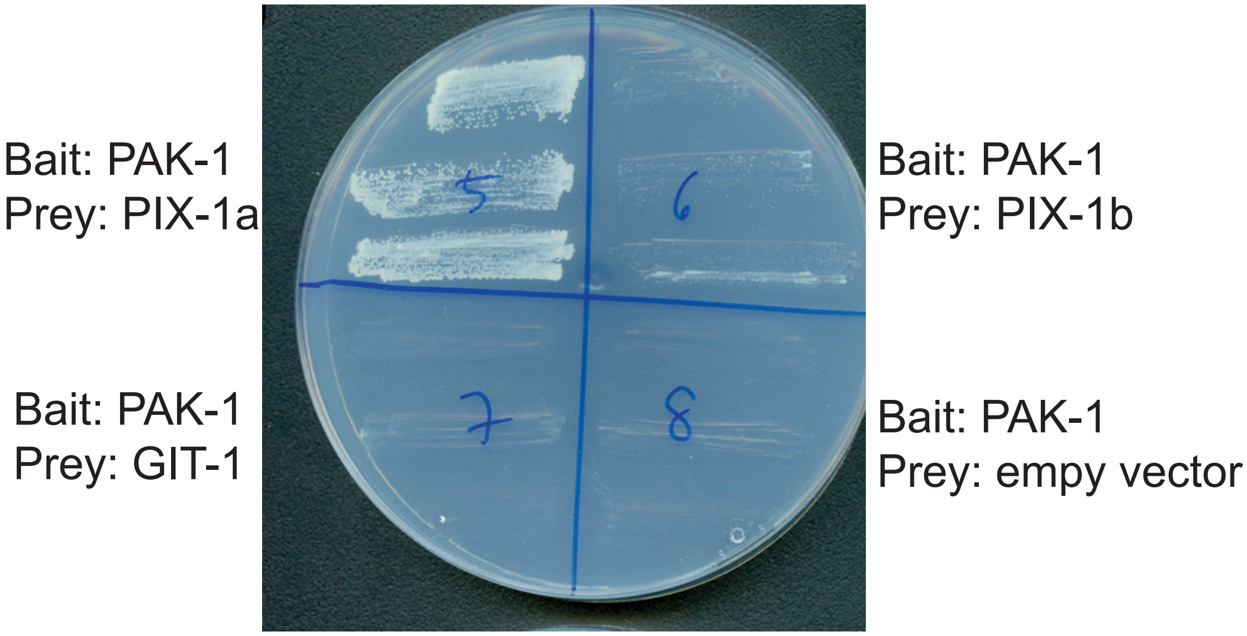

Supplementary Figure 7. Yeast two-hybrid assays showing that full length PAK-1 interacts specifically with full length PIX-1a but not full length PIX-1b or full length GIT-1. Yeast colonies containing the indicated bait and prey plasmids were streaked on -Histidine + 2mM 3AT plates, and incubated at 30 degrees for 3 days. For reproducibility, as indicated, three indepenent yeast colonies were streaked out for each experiment, and they show the same result.

Supplemental Table 1. DH domain proteins in *C. elegans* muscle

C. elegans **protein**	**human ortholog**	**notes**
CGEF-1	MCF2 & MCF2L	
ECT-2	ECT2	
EPHX-1	ARHGEF16	
EXC-5	FGD2 & FGD4	
FRM-3	FARP1 & FARP2	
OSG-1	ARHGEF17	
PIX-1	β-PIX	Rac GEF; localized to M-lines, dense bodies & MCBs
RHGF-1	ARHGEF11	
RHGF-2	PLEKHG5	
SOS-1	SOS1	
TAG-52	ARHGEF39	
TIAM-1	TIAM2	Rac GEF
UIG-1	PLEKHG1	Cdc42 GEF; localized to dense bodies
UNC-73	KALRN	Rac GEF & Rho GEF domains
UNC-89	obscurin	Rho GEF; localized to M-lines
VAV-1	VAV1 & VAV2	
Y37A1B.17	DNMBP	

Supplementary Methods

Yeast two-hybrid assays shown in Supplementary Figure 7. Full length cDNA sequences for PAK-1, GIT-1, PIX-1a and PIX-1b were created by PCR amplification from a cDNA library pool (RB2) using the following primers:

PIX-1a
Pix-1-1: GCGCCCGGGATGGAACCTGGTTCGGGTTCT
Pix-1-3: CGCGTCGACAACCATCGTTAGAGTGAATTC
Pix-1-4: GCGCCCGGGGAATTCACTCTAACGATGGTT
Pix-1-5: CGCGTCGACTTACAAGTCAAACGAGTTGATC

PIX-1b
Pix-1-1: GCGCCCGGGATGGAACCTGGTTCGGGTTCT
Pix-1-2: CGCGTCGACTCACTTCTTCGAGCTTTTTCTTTTG

PAK-1
Pak-1-1: GCGCCCGGGATGAAAGCTTTCTCATCGTATG
Pak-1-2: CGCCTCGAGTCAGCAGGAGTTTCACCATGG
Pak-1-3: GCGCCCGGGACCGCCACCGGTCCCCCATGG
Pak-1-4: CGCCTCGAGTTATGAGTTGCTAGCTTCGGC

GIT-1
Git-1-1: GCGCCCGGGATGTACACAGCAGAGGCGCTTG
Git-1-2: CGCCTCGAGATGCGATGTGAAAGTTGGCCA
Git-1-3: GCGCCCGGGCTAGAGATGCGGGGCATGGCCA
Git-1-4: CGCCTCGAGTCAACAAAATTCCATGATAATC

Except for PIX-1b, the full length cDNAs were created by 2 steps. For example, Pix-1-1 was used with Pix-1-3 to create the 5' half of PIX-1a, and Pix-1-4 and Pix-1-5 was used to create the 3' half of PIX-1a. Each half was cloned into pBluescript and error-free clones selected after DNA sequencing. The two halves of each cDNA were ligated together to create full-length cDNAs. The inserts from these clones were excised using restriction enzymes and ligated into pGBDU-C1(bait plasmid) and pGAD-C1 (prey plasmid). The procedure for performing the yeast two-hybrid assay was described in Miller et al. (2006)[1].

Supplementary References

1. Miller, R.K., Qadota, H., Landsverk, M.L., Mercer, K.B., Epstein, H.F. & Benian, G.M. UNC-98 links an integrin-associated complex to thick filaments in Caenorhabditis elegans muscle. *J. Cell Biol.* **175**, 853-859 (2006).
